# Single-molecule dataset (SMD): a generalized storage format for raw and processed single-molecule data

**DOI:** 10.1186/s12859-014-0429-4

**Published:** 2015-01-16

**Authors:** Max Greenfeld, Jan-Willem van de Meent, Dmitri S Pavlichin, Hideo Mabuchi, Chris H Wiggins, Ruben L Gonzalez, Daniel Herschlag

**Affiliations:** Department of Chemical Engineering, Stanford University, Stanford, CA 94305 USA; Department of Biochemistry, Stanford University, Stanford, CA 94305 USA; Department of Statistics, Columbia University, New York, NY 10027 USA; Department of Physics, Stanford University, Stanford, CA 94305 USA; Department of Applied Physics, Stanford University, Stanford, CA 94305 USA; Department of Applied Physics and Applied Mathematics, Columbia University, New York, NY 10027 USA; Department of Chemistry, Columbia University, New York, NY 10027 USA; Department of Biochemistry, B400, Stanford University, Stanford, CA 94305 USA

**Keywords:** Single molecule, Standardized, File format, SMART, ebFRET, SMD

## Abstract

**Background:**

Single-molecule techniques have emerged as incisive approaches for addressing a wide range of questions arising in contemporary biological research [Trends Biochem Sci 38:30–37, 2013; Nat Rev Genet 14:9–22, 2013; Curr Opin Struct Biol 2014, 28C:112–121; Annu Rev Biophys 43:19–39, 2014]. The analysis and interpretation of raw single-molecule data benefits greatly from the ongoing development of sophisticated statistical analysis tools that enable accurate inference at the low signal-to-noise ratios frequently associated with these measurements. While a number of groups have released analysis toolkits as open source software [J Phys Chem B 114:5386–5403, 2010; Biophys J 79:1915–1927, 2000; Biophys J 91:1941–1951, 2006; Biophys J 79:1928–1944, 2000; Biophys J 86:4015–4029, 2004; Biophys J 97:3196–3205, 2009; PLoS One 7:e30024, 2012; BMC Bioinformatics 288 11(8):S2, 2010; Biophys J 106:1327–1337, 2014; Proc Int Conf Mach Learn 28:361–369, 2013], it remains difficult to compare analysis for experiments performed in different labs due to a lack of standardization.

**Results:**

Here we propose a standardized *s*ingle-*m*olecule *d*ataset (SMD) file format. SMD is designed to accommodate a wide variety of computer programming languages, single-molecule techniques, and analysis strategies. To facilitate adoption of this format we have made two existing data analysis packages that are used for single-molecule analysis compatible with this format.

**Conclusion:**

Adoption of a common, standard data file format for sharing raw single-molecule data and analysis outcomes is a critical step for the emerging and powerful single-molecule field, which will benefit both sophisticated users and non-specialists by allowing standardized, transparent, and reproducible analysis practices.

**Electronic supplementary material:**

The online version of this article (doi:10.1186/s12859-014-0429-4) contains supplementary material, which is available to authorized users.

## Background

Single-molecule techniques have proliferated over the past decade [1-4]. Despite the power of these techniques and their widespread use, critical assessment of single-molecule data remains challenging. While there are multiple reasons for this, principal among these are the inherent noise and stochasticity associated with single-molecule events, which contribute substantially to the analysis challenge. To help manage similarly complex data sets generated from a number of techniques used in modern biological research, other fields have adopted standard data file formats, repositories, and analysis approaches. Examples include the PDB file format for structural data; the RCSB PDB repository of biomolecular structures; the NIH GenBank, DDBJ, and EMBL ENA repositories of gene and genome sequences; the NCBI BLAST and Ensembl sequence alignment and analysis tools; and the CNSsolve biomolecular structure determination tool [5-14]. Standardization has been a key part of the development and advancement of these resources and techniques, facilitating data sharing and dissemination. In addition, the transparency of these formats, repositories, and tools encourages critical assessment of data. Individually the effect of these changes is difficult to assess, but cumulatively they contribute to increased reproducibility and reliability of measurements and, as a result, to the growth and widespread adoption of these techniques.

These examples represent important successes that have arisen naturally. However, several institutions and scientific leaders have recently begun to insist on greater transparency in the dissemination and treatment of all types of scientific data [15,16]. While there are many reasons for this desire and need, a number of well-documented instances within the drug discovery industry where the reproducibility of scientific results has been questioned [17-20] has raised awareness that a lack of easy access to raw data (arising from many sources) and a lack of tools for the primary analysis of the data can undermine clear communication of scientific results and can contribute to erroneous conclusions. Such high-profile problems cannot be attributed to any single failing, but a contributing cause is likely a current lack of standardization and control across the numerous measurement techniques that are combined to support these multidisciplinary development efforts [21,22].

Currently there is no standardization in place to unify the common aspects of most single-molecule data sets and to facilitate the use of the sophisticated analysis approaches that are continually being developed [23-32]. We propose the single-molecule dataset (SMD) file structure as a general data format for storing and disseminating single-molecule data. Moreover, we take steps to facilitate this transition by making two previously established data-analysis packages created in independent labs compatible with this format.

### Implementation

**Figure 1:**
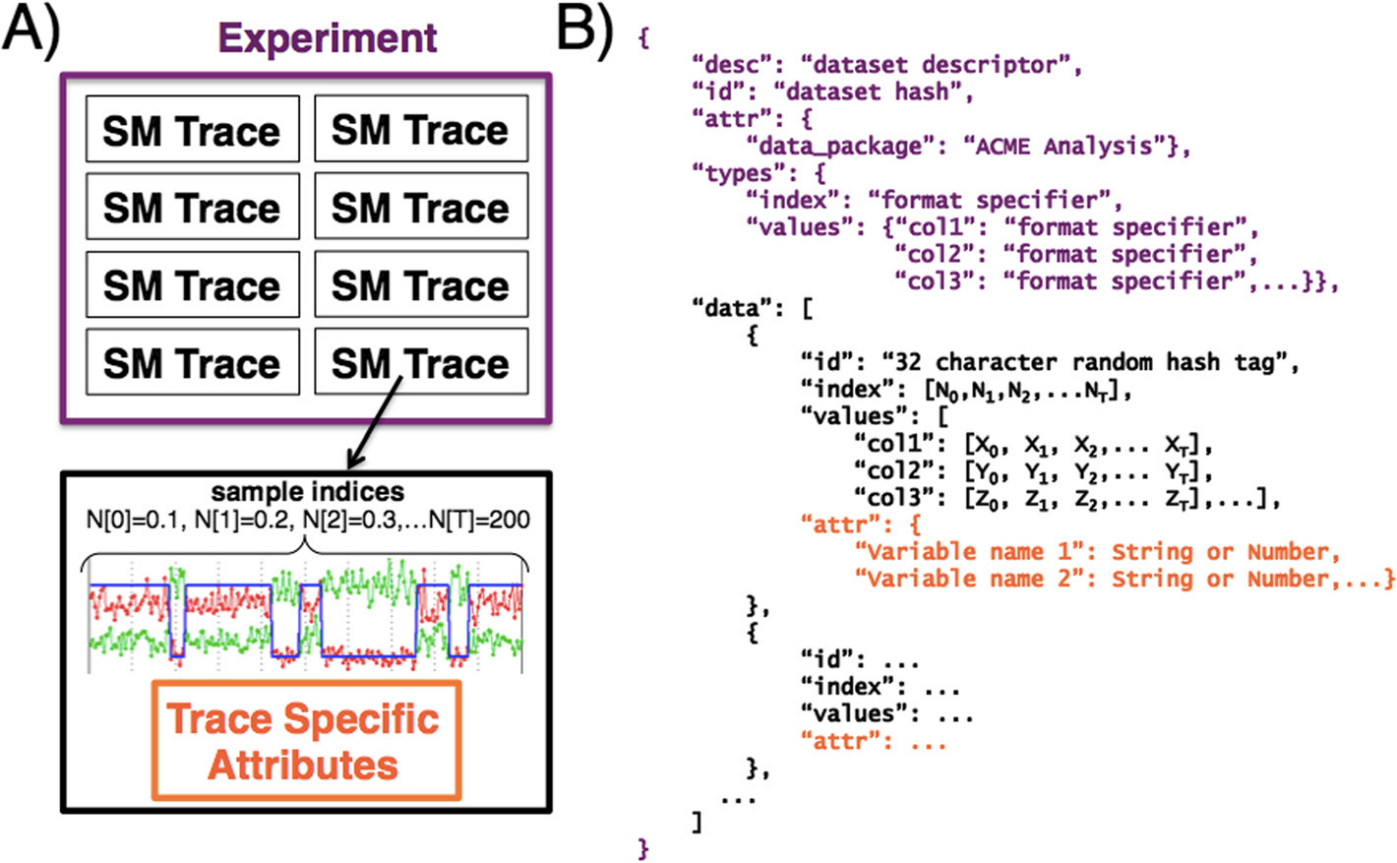
**Structure of SMD. (A)** Cartoon representation of the SMD hierarchy. (Top) Each experiment, represented by the purple rectangle, encompasses the raw data of many single-molecule traces, each represented by a black rectangle. (Bottom) Representation of an individual single-molecule trace within the above experiment. Raw single-molecule data consist of time series data arising from one or more channels. In this example, we depict two channels containing raw data as well as one channel containing an idealized trajectory determined in post-processing. Associated with the raw data of each trace are attributes that are unique to that trace (depicted in orange), such as derived kinetic and thermodynamic parameters obtained from model fitting. **(B)** Representation of the SMD format in JavaScript Object Notation (JSON). The color scheme is used from the cartoon representation in panel (A).

There are many commonalities in how single-molecule data are collected, stored, and analyzed. Figure 1A outlines three unifying relationships that form the basis of the SMD hierarchy. Most single-molecule datasets take the form of time series data (*i.e.*, traces) that are acquired simultaneously from one or more channels during an experiment. While this is not always the rawest form of the data (*e.g.*, a trace can be extracted from a movie recorded using a microscope that can simultaneously monitor many individual molecules), the single-molecule trace unifies many different techniques. At the highest level, a set of single-molecule traces (denoted as black rectangles in Figure 1A, top) are unified by the particular experiment that was used to generate them (denoted as a purple rectangle in Figure 1A, top). Finally, associated with each trace can be experimental information and quantities derived from the analysis of the raw single-molecule data (*e.g.*, inferred kinetic and thermodynamic parameters from model fitting; denoted as orange rectangle in Figure 1A, bottom). The aim of SMD is to encapsulate this hierarchy in a file structure that is independent of any particular programming language, data acquisition platform, or data analysis tool and that is widely compatible with distinct techniques and analysis strategies.

There are many file types that easily accommodate the hierarchy of SMD (HDF5, .MAT, XML, etc.). Indeed, in any high-level analysis package one of these formats is likely to be used. However, to ensure the maximum interoperability between analysis tools, a standard text-based description is advantageous because it allows for straightforward determination of the data fields in a file without any prior knowledge of the specific experiment, data acquisition platform, or data analysis tools used. For interoperability purposes, a SMD object is represented in the widely used JavaScript Object Notation (JSON) format, whose nested structure naturally accommodates the SMD hierarchy.

## Results and discussion

The SMD format aims to strike a balance between defining enough structure to facilitate interoperability of software packages and exchange of data between groups and providing enough flexibility to accommodate data associated with different experimental techniques and analysis use cases. The most important assumption we make is that the dataset holds traces with a fixed set of channels (*e.g.*, raw measurements, post-processed time series, inferred kinetic trajectories, etc.) that are annotated by some set of attributes (*e.g.*, pre-processing settings, fitted model parameters, etc.). The attributes may be quite specific to the type of experiment and analysis performed, but the channel values themselves should in general be suitable to visualization and analysis with different software packages. Figure 1B outlines how the three components of SMD are structured in the JSON notation (the top level is depicted in purple, raw data in black, and trace-specific parameters in orange). Each trace contains four fields. The *values* field stores the trace data where each data type is specified by a descriptive tag. The *index* field contains a list of row labels for the trace (typically measurement acquisition times). Any other trace-specific annotations (*e.g.*, pre-processing settings, fitted model parameters, etc.) are placed in the *attr* field. Finally the *id* field is used to store a 32 digit hexadecimal number generated by running the MD5 algorithm on the data for each trace. The list of traces is itself stored in the *data* field of an outer top-level structure, which itself has a dataset-specific *id* (generated by running the MD5 algorithm on the entire data structure) field as well as an *attr* field that holds top-level annotations or summary statistics that apply to the dataset as a whole (*e.g.*, experimental conditions, time and date of acquisition, averaged model parameters, etc.) and a *desc* field that contains a string describing the data set. Additionally, the dataset-specific *types* specifies the data type for each instance of data being stored in each set of *values.* A full description of the SMD specification is provided in the Additional file 1.

To facilitate the design and adoption of SMD we made the ebFRET [31,32] and SMART [29] single-molecule data analysis packages and visualization tools compatible with the SMD file format. We note here that ebFRET is a descendent of the previously released vbFRET [28,30] data analysis package. We also provide a number of tools for the basic support and validation of SMD files in both Matlab™ and Python packages. Full documentation of SMD and these tools is available at https://smdata.github.io.

The collaboration that resulted in SMD enabled many details that are important for ensuring generality to be implemented. The ebFRET and SMART data analysis packages were developed independently from one another and as a result have significantly different functionalities and work flows. The ability of SMD to easily accommodate these packages with multiple graphical interfaces and distinct outputs provides a strong indication that SMD will be able to accommodate the needs of many researchers.

## Conclusions

Adoption of SMD or, as needed, a different format that encapsulates generalities not anticipated at this time, is an important step for the realization of the full potential of single-molecule measurements by and for a broad scientific community. Although it will require some discipline for researchers to abide by (or “follow”) a common set of standards, the potential long-term benefits are hard to overstate. Standardization will help facilitate the transfer of information among different labs by ensuring that a minimal structure and set of information are present. In turn, this information sharing will facilitate further critical assessment (*e.g.*, data quality, error assessment, and reproducibility) and reanalysis of single-molecule datasets, important steps in extracting the most from complex but information-rich single-molecule data. Moreover, adoption of a common data standard could help facilitate the creation of a repository for single-molecule data (analogous to the RCSB PDB repository of biomolecular structures), which would enable a high degree of transparency and would ensure that data obtained now yields further insights in years to come. We are hopeful that the flexibility of SMD can easily accommodate the needs of current researchers and that it will enable researchers to reap the benefits that accompany widely adopted standardization.

### Availability and requirements

**Project name:** Single-molecule dataset (SMD)

**Project home page:**https://smdata.github.io

**Operating system:** Platform independent

**Programing Languages:** Support provided for Matlab™ and Python, but SMD is not tied to any particular programing language.

**Other requirements:** none

**Licenses:** creative commons

**Any restrictions to use by non-academics:** none

## Additional file

Additional file 1:
**Technical documentation for the SMD format and supporting Matlab™ and Python packages.**
