# Supplementary material for: Single-molecule dataset (SMD): a generalized storage format for raw and processed single-molecule data
Source: BMC Bioinformatics. 2015 Jan 16;16(1):3. doi: 10.1186/s12859-014-0429-4 (PMC4384321; doi:10.1186/s12859-014-0429-4)
Supplement: Additional file 1: — Technical documentation for the SMD format and supporting Matlab™ and Python packages. [file 12859_2014_429_MOESM1_ESM.docx]

**Single-molecule data file format (SMD): A generalized storage format for raw and processed single-molecule data**

Max Greenfeld^a,b ,‡^, Jan-Willem van de Meent^c‡^, Dmitri S. Pavlichin^d^, Hideo Mabuchi^e^_,_ Chris H. Wiggins^f^, Ruben L. Gonzalez Jr.^g^, and Daniel Herschlag^a,b,h^

Departments of ^a^Chemical Engineering, ^b^Biochemistry, ^d^Physics and ^e^Applied Physics, Stanford University, Stanford, CA 94305

Departments of ^c^Statistics, ^f^Applied Physics and Applied Mathematics, and ^g^Chemistry,

Columbia University, New York, NY 10027

^‡^These authors contributed equally

^g^Corresponding author:

Dept. of Chemistry

Columbia University

New York, NY 10027-3126

212-854-1096 (telephone)

212-932-1289 (fax)

rlg2118@columbia.edu

^h^Corresponding author:

Dept. of Biochemistry, B400

Stanford University

Stanford, CA, 94305-5307

650-723-9442 (telephone)

650-723-6783 (fax)

herschla@stanford.edu

Additional email addresses of authors:

Max Greenfeld: max.greenfeld@gmail.com

Jan-Willem van de Meent: janwillem.vandemeent@gmail.com

Dmitri S. Pavlichin: dmitrip@stanford.edu

Hideo Mabuchi: hmabuchi@stanford.edu

Chris H. Wiggins: chris.wiggins@columbia.edu

**Comprehensive Description of SMD**

The single-molecule data format (SMD) relies on the JavaScript Object Notation (JSON) format to enforce the structure. That is, every SMD format must be a valid JSON file. The JSON format is a highly generalized format that is capable of storing virtually any type of data. The SMD format simply imposes an order to how the JSON format should be used for storing single-molecule data.

Figure S1A displays the layout of the SMD format. There are three levels of hierarchy on the SMD format. The top layer of an SMD file stores an arbitrary number of uniquely identified ‘experiments.’ This allows an investigator or a software package using the SMD format a large amount of flexibility to combine datasets with potential diverse attributes defining those datasets in one file type. The second layer of the SMD format is a group that is comprised of *traces* that have a large number of attributes in common. While users of the SMD format can choose to use this layer of organization as they see fit, a natural way to consider this level is that it would be composed of many traces arising from the same experiment. The third layer is composed of parameters that are derived from, or are unique to, a particular trace.

The top-level structure of the SMD format contains four fields:

- **desc.** This field serves to provide a simple descriptor of the data set contained herein.
- **id.** This field serves as a unique identifier for the particular set of traces that are grouped in this data structure. By default, a MD5 algorithm is used to generate a 32 digit hexadecimal number that is practically unique. This helps to ensure that when datasets generated at different times are combined, it remains easy to track the source of each dataset.
- **attr.** The attributes field stores information related to a particular group of traces. This could be information such as the day the experiment was completed, the exact experimental conditions, or any other information that relates to the data set as a whole.
- **types.** Holds type identifiers for the **index** and **values** fields. Each field of data being stored in the **values** field should be specified here. These identifiers are ‘bool’, ‘float’, ‘double’, ‘int’, ‘long’ and ‘string’.
- **data.** Holds a list of entries for each trace, which themselves contain a set of fields:
- **id.** Holds a trace-specific identifier. By default a MD5 hash is of the **values** structure is used.
- **index.** This field contains a list of row labels for the **values** matrix, which typically hold the measurement times. This field should have the same length as the data in the **values** field.
- **values.** This field contains the actual single-molecule data. Most simply, each data type being used is stored in a field with a descriptive name (*e.g.*, channel1). While this is primarily intended to store raw single-molecule data, it could equally well be used to store window-averaged data, thresholded data, fits of the data or an arbitrary number of other series data.
- **attr.** This attributes field has much the same role as the top-level attributes field, but is specific to this particular trace. Within this data field a user can store any additional information they are interested in storing. This could be anything from a kinetic or thermodynamic parameter algorithmically determined for a particular trace to an observation of that particular trace that an experimentalist wants to note for future reference.

**SMD Toolboxes for Matlab™ and Python**

To facilitate the adoption of the SMD format, Matlab™ and Python toolboxes were created. These toolboxes allow SMD files to be generated and validated as well as simple functions such as sorting and merging to be performed. The toolboxes can be found at the following link: https://smdata.github.io/.

Figure S2A depicts how data fields should be populated in a Matlab™ structure to enable conversion to an SMD file using the SMD Matlab™ toolbox. The fields have the same names and meaning as described above. For data formatted in this manner, the following functions in the SMD toolbox can be used to manipulate these structures and crate SMD files. In addition to a basic version of Matlab™, this toolbox requires JSONlab, a freely available toolbox for encoding and decoding JSON files in Matlab™ (see: http://iso2mesh.sourceforge.net/cgi-bin/index.cgi?jsonlab).

**smd.create(data, types, varargin)**: Creates a SMD structure from supplied data.

**smd.write_json(filename, dataset)**: Saves a SMD structure as JSON (.json)or compressed JSON (.json.gz).

**smd.read_json(filename)**: Loads a SMD structure from JSON (.json)or compressed JSON (.json.gz).

**smd.isvalid(dataset)**: Checks if supplied struct is a valid SMD instance.

**smd.filter(dataset)**: Returns a filtered dataset by matching id and attr values, or by applying a custom function with boolean output to each trace.

**smd.merge(data1, data2, ...)**: Returns a merged dataset containing all traces in multiple datasets.

Figure S2B depicts how data fields should be populated in a Python dictionary to enable conversion to an SMD file using the SMD Python toolbox. The fields have the same names and meaning as described above. For data formatted in this manner the following functions in the SMD toolbox can be used to manipulate these structures and crate SMD files. The functionality of the Python toolbox mimics that of the Matlab™ toolbox previously described.

**
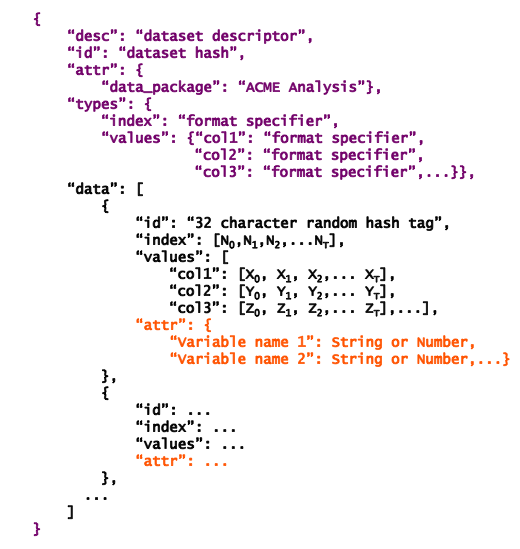
**

**Figure S1:** Generic outline of the SMD format in JSON notation. All fields required by the SMD notation are shown.


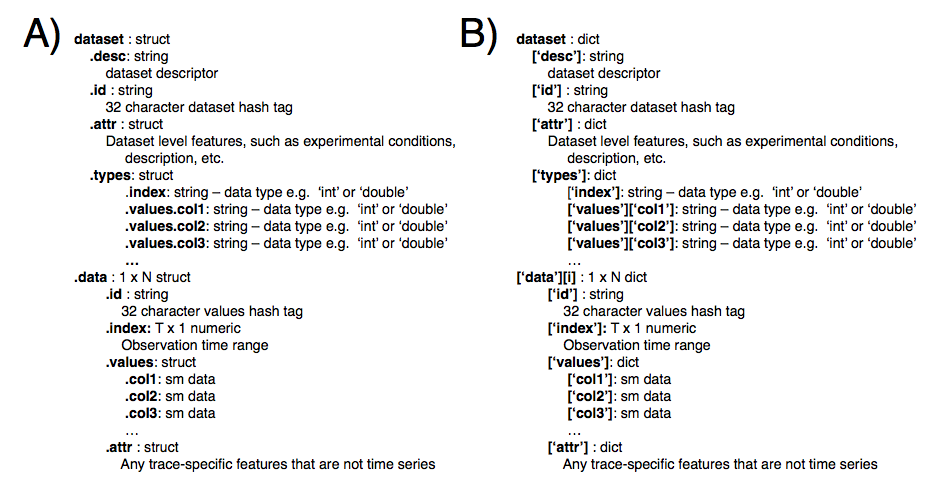


**Figure S2:** Schematic representations of the SMD format. **(A)** Representation of single-molecule data in Matlab™ prior to conversion to the SMD format with the SMD Matlab™ toolbox. **(B)** Representation if single-molecule data in a Python dictionary prior to conversion to the SMD format with the SMD Python toolbox.
